# Human mesenchymal stem cells in spheroids improve fertility in model animals with damaged endometrium

**DOI:** 10.1186/s13287-018-0801-9

**Published:** 2018-02-26

**Authors:** Alisa Domnina, Polina Novikova, Julia Obidina, Irina Fridlyanskaya, Larisa Alekseenko, Irina Kozhukharova, Olga Lyublinskaya, Valeriy Zenin, Nikolay Nikolsky

**Affiliations:** 1 Institute of Cytology, Russian Academy of Sciences, St. Petersburg, Russia; 2 Faculty of Medicine, St. Petersburg State University, St. Petersburg, Russia

**Keywords:** Endometrial mesenchymal stem cells, Cell spheroids, Asherman’s syndrome, Animal model

## Abstract

**Background:**

Asherman’s syndrome (AS) is one of the gynecological disorders caused by the destruction of the endometrium. For some cases of AS available surgical methods and hormonal therapy are ineffective. Stem cell transplantation may offer a potential alternative for AS cure.

**Methods:**

Human endometrial mesenchymal stem cells (eMSC) organized in spheroids were transplanted in rats with damaged endometrium modeled on AS. Treatment response was defined as pregnancy outcome and litter size.

**Results:**

Application of eMSC in spheroids significantly improved the rat fertility with the AS model. eMSC organized in spheroids retain all properties of eMSC in monolayer: growth characteristics, expression of CD markers, and differentiation potential. Synthesis of angiogenic and anti-inflammatory factors drastically increased in eMSC assembled into spheroids.

**Conclusions:**

Human endometrial mesenchymal stem cells (eMSC) can be successfully applied for Asherman’s syndrome (AS) treatment in the rat model. eMSC organized in spheroids were more therapeutically effective than the cells in monolayer. After transplantation of eMSC in spheroids the pregnancy outcome and litter size in rats with AS was higher than in rats that received autologous rat bone marrow cells. It suggests the therapeutic plausibility of heterologous eMSC in case of failure to use autologous cells.

## Background

Infertility diagnosis defined as a failure to conceive is given approximately to one of every six couples in the population [1]. When a cause of infertility exists in a female partner it is referred to as female infertility. The important reason of female infertility is endometrial dysfunction. One of the gynecological disorders caused by the destruction of the endometrium is Asherman’s syndrome (AS). The main cause of AS is repeated or aggressive curettages and/or endometritis [2]. It leads to a loss of functional endometrium and the uterine cavity is obliterated by intrauterine adhesions. AS is characterized by amenorrhea, hypomenorrhea, recurrent pregnancy loss and/or abnormal placentation, including placenta previa and accrete [3]. The incidence of AS varies between 2 and 22% of infertile women [2, 4]. Treatment of AS aims to restore uterine fertility and parturition. The traditional AS therapy is hormonal medications to regenerate the endometrium or surgical resection of adhesions combined with insertion of intrauterine devices or adhesion barriers to prevent recurrent adhesions [5, 6]. However, severe damage of the basal layer of endometrium causes loss of the local endometrial progenitor cells and leads to regeneration failure and repeated adhesion formation [7–9]. Stem cell transplantation may offer a potential cure for severe AS.

Administration of mesenchymal stem cells (MSC) for cell therapy of various diseases is under extensive research. The MSC possess high proliferative activity and are capable of differentiating into different cell types. The main advantage of MSC for clinic application is the possibility to use the patient’s own cells (autologous MSC) for transplantation. It reduces the risks of rejection and undesirable immunological reactions. For a long time bone marrow has been the main MSC source. To date, MSC have been established from adipose tissue [10], umbilical cord blood [11], amniotic fluid [12], and endometrium [13, 14] as well as other sources [15].

However, the methods of receiving cellular material from bone marrow and adipose tissue are painful and can be dangerous for donors; cells from umbilical cord can be received only once in life (at the birth).

Human endometrium composed of endometrial glands and outlined with stroma is a dynamic tissue undergoing about 400 cycles of regeneration, differentiation, and shedding. It was found that endometrium fragments in the menstrual blood are the source of stem cells. Menstrual blood is noninvasive and easily available material for isolation of endometrial MSC (eMSC). Their high proliferation activity during long-term cultivation, genetic stability [16], lack of tumorigenicity, and low immunogenicity [17] make the eMSC from the menstrual blood a promising source of stem cells for future clinical applications.

It is believed, that mesenchymal stem cell therapy promotes healing by manipulating the local environment that enhances the function of host cells [18]. A major impediment in the cell-based therapy is a high death rate and poor engraftment of cells in sites of damage [19]. Many efforts are aimed to overcome this challenge and improve survival of transplanted cells. An approach to improve the efficacy of MSC-based therapy is MSC preparation as three-dimensional spheroids (3D culture). It was demonstrated that MSC in spheroids increased transplanted cell survival and efficacy of stem cell therapy [20, 21].

In this study, we examined the therapeutic potential of human eMSC in the treatment of endometrium injury in the rat model of Asherman’s syndrome and compared the therapeutic effect of these cells maintained in monolayer and spheroids.

## Methods

### Cells

eMSC used in our experiments were obtained from the MSC collection of the Department of Intracellular Signaling and Transport of the Institute of Cytology RAS, Russia. These cells were established from menstrual blood with endometrium fragments obtained from healthy woman at the age of 27 years [22]. The cells were plated in 6-cm Petri dishes (Corning, Corning, NY, USA) in DMEM/F12 medium with 10% fetal calf serum (FCS) (HyClone, Logan, UT, USA), 1% antibiotic–antimycotic mixture, and 1% glutamaxandand cultivated for 3–7 days. The cells were subcultured with EDTA/trypsin solution.

Human embryonic cell line (ESC) C910 [23] was obtained from the Department of Intracellular Signaling and Transport of the Institute of Cytology RAS, Russia. These cells was maintained in mTeSR1 medium (Stem Cell Technologies, Vancouver, BC, Canada) on Petri dishes covered with mitomycin-treated eMSC as feeder layer. The cells were subcultured mechanically.

### Spheroid formation

Spheroids were formed from eMSC at the 4–6th passages using the hanging drop technique [18]. 5000–7000 cells per 35 μL were placed in drops on the cover of 96-well plates and inverted then over single wells. Cells spontaneously aggregated in hanging drops for 48 h were then transferred for 24 h in dishes coated with 2-hydroxyethyl methacrylate (HEMA*;* Sigma-Aldrich, St. Louis, MO, USA). Single cell suspension was obtained by spheroid treatment with 0.05% trypsin/EDTA and used to monitor spheroid cell properties.

### Immunophenotyping

Immunophenotyping (CD marker expression) of monolayer eMSC and eMSC spheroids was performed with an Epics XL flow cytometer (Beckman Coulter, Brea, CA, USA). The single cell suspension was obtained using 0.05% trypsin/EDTA. 1 × 10^6^ cells were suspended in 1 mL of PBS with 5% FCS. FITC-conjugated antibodies to CD34, CD 44, CD45, CD90, CD 146, HLA-1, and phycoerythrin (PE)-conjugated antibodies to CD73, CD105, CD140b, and HLA DR were applied.

### Adipogenic differentiation

2 × 10^4^ cells/cm^2^ were seeded in Petri dishes coated with 0.1% gelatin (Sigma-Aldrich, St. Louis, MO, USA). When the cells reached about 80% confluence, 1 mM dexamethasone (Sigma-Aldrich, St. Louis, MO, USA), 0.5 mM isobutyl-methyl-xanthine (IBMX; Sigma-Aldrich, St. Louis, MO, USA), 10 μg/mL human recombinant insulin (Sigma-Aldrich, St. Louis, MO, USA) and 100 mM indomethacin were added. In this medium, the cells were differentiated for 3–5 weeks with a half volume of the medium changed every 2–3 days. Lipid drops were visualized with Oil Red staining (Sigma-Aldrich, St. Louis, MO, USA) according to the manufacturer’s instructions. Adipogenic differentiation was also tested with RT-PCR. Primers for adipogenic differentiation are shown in the Table 1.

**Table 1:** Primer sequences for control and target genes and q-PCR conditions

Symbol	Primer sequence	Amplification conditions	PCR product size (bp)	Accession number	Reference
TSG-6	S: GATGGATGGCTAAGGGCAGAGT-3′	93 °C, 20 s, 61 °C, 20 s 72 °C 30 s	208	NM_007115.3	Liu et al. 2016 [44]
	AS: TCATTTGGGAAGCCTGGAGATT-3′				
EP2	S: 5-CCACCTCATTCTCCTGGCTA-3	93 °C, 20 s, 62 °C, 20 s 72 °C 30 s	216	NM_000956.3	Kunisch et al. 2009 [45]
	AS: 5-CGACAACAGAGGACTGAACG-3				
HGF	S: 5′-CTCACACCCGCTGGGAGTAC-3′	93 °C, 20 s, 62 °C, 20 s 72 °C 30 s	104	XM_011516115.2	Jankowski et al. 2003 [46]
	AS: 5′-TCCTTGACCTTGGATGCATTC-3′				
RUNX2	S: GCCTTCAAGGTGGTAGCCC-3′	93 °C, 20 s, 62 °C, 20 s 72 °C 30 s	67	XM_017011396.1	Shafiee et al. 2011 [47]
	AS: CGTTACCCGCCATGACAGTA-3′				
OPN	S: 5′-TTGCAGCCTTCTCAGCCA-3′	93 °C, 20 s, 62 °C, 20 s 72 °C 30 s	76	NM_001251830.1	Bahrambeigi et al. 2012 [48]
	AS: 5′-GGAGGCAAAAGCAAATCACTG-3′				
FABP4	S: 5′-ATGGGATGGAAAATCAACCA-3′	93 °C, 20 s, 59 °C, 20 s 72 °C 30 s	87	NM_001442.2	Ponnaiyan et al. 2014 [49]
	AS: 5′-GTGGAAGTGACGCCTTTCAT-3′				
GAPDH	S: 5′-GACTCATGACCACAGTCCATGC-3′	93 °C, 20 s, 67 °C, 20 s 72 °C 30 s	112	NM_001289746.1	Liang et al. 2015 [50]
	AS: 5′-AGAGGCAGGGATGATGTTCTG-3′				
NANOG	S: 5′-CAAAGGCAAACAACCCACT-3′	93 °C, 30 s, 60 °C, 30 s 72 °C 30 s	427	NM_024865.2	Kozhukharova et al., 2009 [23]
	AS: 5′-CTGGATGTTCTGGGTCTGGT-3′				
OCT4	S:5′-AGCCCTCATTTCACCAGGCC-3′	93 °C, 30 s, 63 °C, 30 s 72 °C 30 s	456	NM_002701.5	Liedtke et al., 2007 [51]
	AS:5′-TGGGACTCCTCCGGGTTTTG-3′				
SOX2	S:5′ GCGCATGGACAGTTACGC-3′	93 °C, 30 s, 60 °C, 30 s 72 °C 30 s	276	NM_003106.3	Koshkin et al. 2016 [52]
	AS: 5′ TCGGACTTGACCACCGAAC-3′				
ACTIN	S:5′-GCCGAGCGGGAAATCGTGCGT-3′	93 °C, 30 s, 70 °C, 30 s 72 °C 30 s	507	NM_001101.3	Alekseenko et al., 2012 [53]
	AS:-5’-CGGTGGACGATGGAGGGGCCG-3′				

### Osteogenic differentiation

2 × 10^4^ cells/cm^2^ were seeded in Petri dishes coated with 0.1% gelatin. After the cells reached 100% confluence 100 nM dexamethasone, 10 mM β glycerol phosphate, and 0.2 mM ascorbate- 2- phosphate were added. In this medium, the cells were differentiated for 3–5 weeks with a half volume of the medium changed every 2–3 days. Then, the cells were fixed with 70% cold ethanol for 1 h and stained with Alizarin Red, pH 4.1 (Sigma-Aldrich, St. Louis, MO, USA). Osteogenic differentiation was also tested with RT-PCR. Primers for osteogenic differentiation are shown in the Table 1.

### Decidual differentiation

eMSC were seeded in 24-well plates in the growth medium. After the cells reached 80% density, the medium was exchanged for serum-free medium for 24 h. The medium was then exchanged for the medium with 2% FCS and 1 mM 8-Br-cAMP (Sigma-Aldrich, St. Louis, MO, USA) and then was changed every 3 days. Control cells were cultivated in the same medium but without 8-Br-cAMP. After 7 days, the medium from control and differentiated cells was collected for prolactin and insulin-like growth factor binding protein-1 (IGFBP-1) testing. The assay was done with ELISA kits for prolactin and IGFBP-1 quantitative measurements (Sigma-Aldrich, St. Louis, MO, USA). The amount of prolactin and IGFBP-1 was normalized to the total cell protein determined with the Bradford method.

### SA β-galactosidase staining

Cell senescence, was assayed with Senescence β-Galactosidase Staining Kit (Cell Signaling, Danvers, MA, USA). Cells were plated in 3.5-cm Petri dishes. 2–3 days after seeding, the medium was discarded and cells were washed with PBS, and fixed with formaldehyde/glutaraldehyde mixture at room temperature for 10–15 min. Fixed cells were thoroughly washed with PBS and incubated with staining solution at 37 °C without CO_2_ for 8 h. The pH value of staining solution should be kept at a level of 6.0. The staining pattern was visualized under the light microscope. Senescent cells were identified by the blue color in cytoplasm (SA*-*β*-*Gal-positive).

### RT-PCR and qRT-PCR assays

To analyze gene expression, total RNA was isolated with RNesy Micro Kit (Qiagen, Valencia, CA, USA) according to the manufacturer’s instructions. RNA was quantified in the NanoDrop ND-1000 Spectrophotometer (NanoDrop Technologies, Inc., Wilmington, DE, USA). cDNA was obtained by reverse transcription of RNA using the Revert Aid H Minus First-Strand cDNA Synthesis Kit (Fermentas, Vilnius, Lithuania) according to the manufacturer’s instructions. It was subsequently amplified with specific primers, using DreamTaq™ PCR Master Mix (2x) (Thermo Fisher Scientific, Waltham, MA, USA) with CycloTemp amplificator. The electrophoresis of amplified products was performed in 2% agarose gel with TAE buffer and ethidium bromide. 100 kb DNA ladder (Fermentas, Vilnius, Lithuania) was used as molecular weight markers. Amplified products were visualized in UV light (302 nm) with Transilluminator and registered with a digital Canon camera (Canon, Tokyo, Japan). For qRT-PCR cDNA was amplified with specific primers, using EvaGreen® dye (Biotium, Fremont, CA, USA) and DreamTaq™ PCR Master Mix (2X) (Thermo Fisher Scientific, Waltham, MA, USA) in the Bio-Rad CFX-96 real time system (Bio-Rad, Hercules, CA, USA), according to the kit’s enclosed protocol. Expression of target genes was normalized to *gapdh* gene. Primers and reaction conditions are presented in the Table 1. All amplifications were performed in triplicates. Experiments were repeated at least three times.

### Animals

All experiments were performed with Wistar rats, weight 200–250 g. The animals were maintained in the designated animal care facility with free access to tap water and food. All experimental procedures with animals were performed according to the Institutional Guidelines for the Care and Use of Laboratory Animals. All studies on animals were performed after approval by the Institutional Animal Care and Use Committee of Institute of Cytology RAS (Assurance Identification number F18–00380).

### Harvesting of rat bone marrow

Rat bone marrow (BM) was flushed from the femurs and tibias of adult Wistar females with sterile PBS. The cell suspension was filtered through sterile 70-mM Nitex mesh (Becton, Dickinson and Company, Franklin Lakes, NJ, USA) and used as transplantation material.

### Animal modeling of the Asherman’s syndrome

Adult albino Wistar rat females weighing 200–250 g were used in experiments. Vaginal cytology was performed to evaluate the stage of estrous cycle. A sterile swab was moistened with saline and rotated against the vaginal wall to obtain vaginal cells. Vaginal smears were visualized with the light microscope. Only animals in diestrus were used. Animals were anesthetized by intramuscular injection of Zoletil 100 (Virbac, Carros, France) in a dose 5 mg/kg weight; surgical manipulations were done under aseptic conditions. The animals were fixed in supine position, and the inferior abdomen was sterilized and shaved. An incision of approximately 2.5 cm was made into the inferior abdomen through the skin and underlying layers and uterus horns were pulled out.

0.3 ml of 95% ethanol were injected into both uterine horns and kept for 3 min. Uterine horns cavities were washed with 0.5 ml of PBS solution. Then, the uterus was put back into the abdominal cavity and the abdominal muscles and skin were sutured. About 100 female rats underwent the induction of modeled Asherman’s syndrome (AS). They were randomized into different groups, differing in transplantation material (rat BM, eMSC monolayer, eMSC spheroids) and delivery mode (vein or intrauterine injection). eMSC spheroids were transplanted only into the uterus. Intravenous administration entails the cell trapping in lungs with a high risk of embolism. Animals were subjected to cell therapy 72 h after the uterine injury. Each rat received 0.2 mL PBS (control) or 0.2 mL cell suspension in PBS. Cell suspension contained 10^7^ cells for the vein injection and 10^6^ cells for intrauterine injection. Vein injection was done via the tail vein. For intrauterine transplantation the animals were fixed in the dorsal position. Double sections of skin and muscles were done 1.5 cm laterally to the vertebrae. Uterus horns were pulled out very carefully to avoid any traumatization and intrauterine injections were performed. Three estrous cycles after AS induction the females were mated with Wistar males for a period of 3 months, and the number of pregnant rats and litter size were recorded. The control AS model rats were sacrificed before mating, and the uterine horns were taken for histological examination.

### Histology

Frozen 10-μm sections of uterine horns were made. Slides were fixed in ethanol/methanol mixture for 2 min at −20 C° and stained with hematoxylin and eosin (H&E) or Trichrome. Structural alteration in uterus, fibrosis, and presence of inflammation and necrosis areas were assessed by the light microscopy.

### Statistical analysis

The data are presented as the mean ± standard deviation (SD), when indicated. Difference in pregnancy rate was analyzed by Fisher’s exact test. The difference in the number of pups was tested by Kruskal-Wallis H-test (non-parametric ANOVA) followed by post hoc pairwise comparison using Mann-Whitney *U* test with Bonferroni correction when appropriate. All other data were treated using Student’s *t* test (two-tailed). Data were analyzed with IBM SPSS Statistics v.22 software (IBM Corp., Armonk, NY, USA). Differences were considered significant at *p* < 0.05.

## Results

### Stem properties of eMSC spheroids

**Fig. 1:**
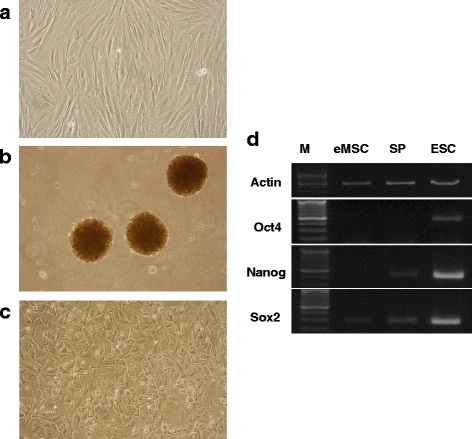
Stem properties of eMSC spheroids. **a** eMSC monolayer; **b** eMSC in spheroids; **c** eMSC after spheroids dissociation, **d** pluripotent gene expression in MSC spheroids and human embryonic stem cells (ESC). *eMSC* endometrial mesenchymal stem cells

**Fig. 2:**
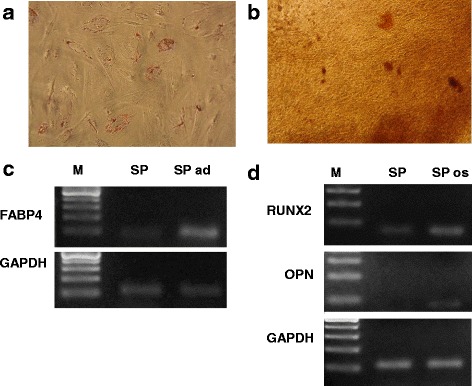
Multipotency of eMSC spheroids. **a** adipogenic differentiation of eMSC spheroid; **b** osteogenic differentiation of eMSC spheroids; **c**, **d** expression of genes involved in adipogenic (**c**) and osteogenic (**d**) differentiation

Figure 1 shows eMSC in monolayer and spheroids. Maintenance of eMSC in spheroids did not modify their stem cells properties. Being dissociated, they display a fibroblast-like morphology although they are initially smaller and less adhesive than cells permanently maintained in monolayer conditions (Fig. 1c). Serial subculturing of spheroid-derived cells attenuates the difference in spheroid and monolayer cell morphology. eMSC in spheroids as their monolayer counterparts express CD140b, CD105, CD73, CD90, CD44, HLA-1, lack CD34, CD45, and HLA DR (class II) (Table 2). Unlike monolayer cells eMSC spheroids were not CD146-positive. Cells in spheroids exhibited upregulation of pluripotency genes (*Nanog, Sox2*) but their expression is weaker than in human embryonic stem cells ESC (Fig. 1d). eMSC spheroids retain multipotency. They stay able to differentiate into adipocytes (Fig. 2a) and osteoblasts (Fig. 2b) visualized by staining with specific dyes and RT-PCR expression of FABP4 gene-regulated adipogenesis (Fig. 2c) as well as osteoporin (OPN) and RUNX2 (a key transcription factor) involved in osteoblast differentiation (Fig. 2d).

**Table 2:** CD expression in eMSC monolayer and spheroids

CD marker	eMSC monolayer	eMSC spheroids
CD34	0.00%	0.00%
CD 44	91.50%	91.00%
CD 45	8.10%	0.00%
CD 73	96.80%	95.10%
CD 90	91.40%	91.00%
CD 105	95.40%	71.70%
CD 140b	90.30%	75.90%
CD 146	58.30%	0.30%
HLA-1	91.60%	91.00%
HLA-DR	0.00%	0.00%

### Proliferation and senescence of eMSC spheroids

**Fig. 3:**
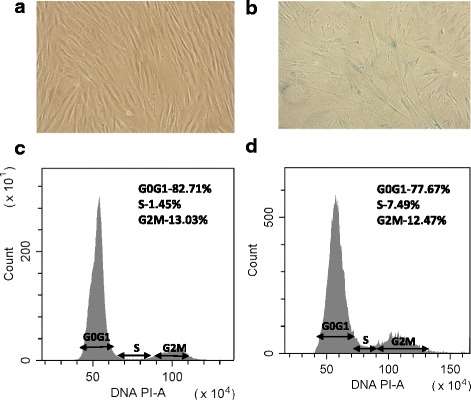
Proliferation and senescence of eMSC spheroids. **a**, **b** β-galactosidase activity in eMSC after spheroids dissociation and subculturing for 2 (**a**) and 15 (**b**) passages; **c**, **d** cell cycle analysis of eMSC spheroids (**c**) and monolayer cells (**d**)

It was reported that quiescent cells are more favorable for transplantation [24]. Cell cycle analysis of eMSC spheroids and monolayer cells used for transplantation was analyzed with flow cytometry (Fig. 3). It was found that most cells in spheroids are in G0/G1 stage (83%). eMSC maintained under routine conditions were also mostly in G0/G1 stage, because they were used from dense cultures when cells stopped dividing (78%). However, cell dissociated from spheroids continued to proliferate. During early passages, the cells have a fibroblast-like shape and form a monolayer at high density. The proliferation rate of spheroid-derived MSC gradually declines during subculturing. At late passages (14–16), the cells enlarged, flattened, were unable to generate monolayers. They became SA-β-gal-positive, which is an indication that cells entered into the replicative senescence phase (Fig. 3).

### Decidualization of spheroid eMSC

A key stage during pregnancy is the endomentrial remodeling in preparation for embryo implantation defined as the decidualization process. It was found that eMSC transplanted into the uterus stimulated the development of decidual tissue in rats [25]. Decidualization potential of menstrual blood-derived mesenchymal stem cells in vitro has been reported by other researchers [26]. We demonstrated that eMSC exhibited higher capacity for differentiation into decidual cells than MSC from adipose tissue and bone marrow [27].

**Fig. 4:**
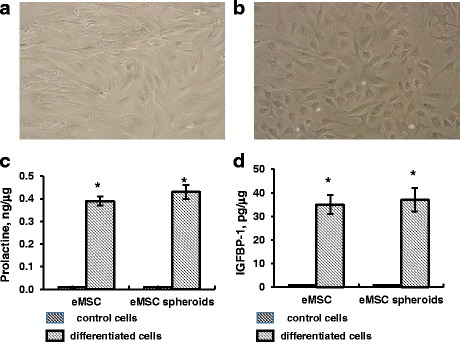
Decidual differentiation of eMSC spheroids. **a** eMSC spheroids, control cells; **b** eMSC spheroids, treated with 1 mM 8-Br-cAMP for 7 days; **c**, **d** ELISA assay of prolactin (**c**) and IGFBP-1 (**d**) secretion of control and induced to decidual differentiation eMSC spheroids and monolayer cells. Data are shown as mean ± SD. Two-tailed Students *t* test was utilized for pairwise comparison. *p < 0.01 vs. control undifferentiated cells. *eMSC* endometrial mesenchymal stem cells, *IGFBP-1* insulin-like growth factor binding protein-1

In this study, we compared the level of decidual differentiation of monolayer and spheroid eMSC (Fig. 4). During decidual differentiation induced by 1 mM 8-Br-cAMP, eMSC fibroblast-like morphology alters. The cells acquire polygonal shape with enlarged nuclei typical for decidual cells (Fig. 4b). Secretion of prolactin and IGFBP-1 secretion are major decidual cell markers. The results of measurements of prolactin and IGFBP-1 secretion are presented in Fig. 4. It is seen that undifferentiated control cells have a low secretion level. The prolactin level drastically increased (*p* < 0.01) in eMSC and eMSC spheroids cultivated with 8-Br-cAMP for 7 days (Fig. 4c). Similar results have been obtained with IGFBP-1 secretion (Fig. 4d). We did not observe the difference in the potential of monolayer and spheroid eMSC to decidual differentiation induction. The level of decidualization of spheroid cells was slightly higher than cells in monolayer, but it was not statistically significant.

### Expression of therapeutic cytokines by eMSC spheroids

**Fig. 5:**
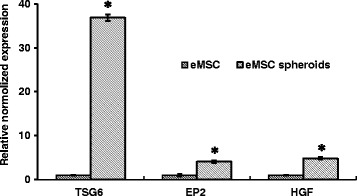
q-RT-PCR assay of TSG6, EP2, and HSF genes in eMSC spheroids and monolayer cells. Data are shown as mean ± SD. Two-tailed Student’s *t* test was utilized for pairwise comparison. *p < 0.01 vs. eMSC in monolayer. *eMSC* endometrial mesenchymal stem cells, *EP2* prostaglandin E receptor, *HGF* hepatocyte growth factor, *TSG-6* tumor necrosis factor-α-induced protein 6

Increasing evidence supports that the therapeutic MSC effect is mediated via the secretion of trophic factors, such as angiogenic, antitumorigenic, pro- and anti-inflammatory molecules. Cell aggregation into spheroids enhances paracrine secretion [28]. In our work, we compared expression of genes encoding these factors (TSG-6, EP2, and HSF) in adherent and spheroid eMSC. Tumor necrosis factor-α-induced protein 6 (TSG-6) is an anti-inflammatory protein, Hepatocyte growth factor (HGF) protein exhibits anti-apoptotic and angiogenic properties, prostaglandin E receptor (EP2) has immunomodulatory function. Figure 5 demonstrates that HGF and EP2 expression in eMSC spheroids enhances about five times whereas TSG6 expression increased by more than 30 times. It shows that eMSC organization into spheroids stimulates the cell secretory function.

### Histological analysis of uterus injury

**Fig. 6:**
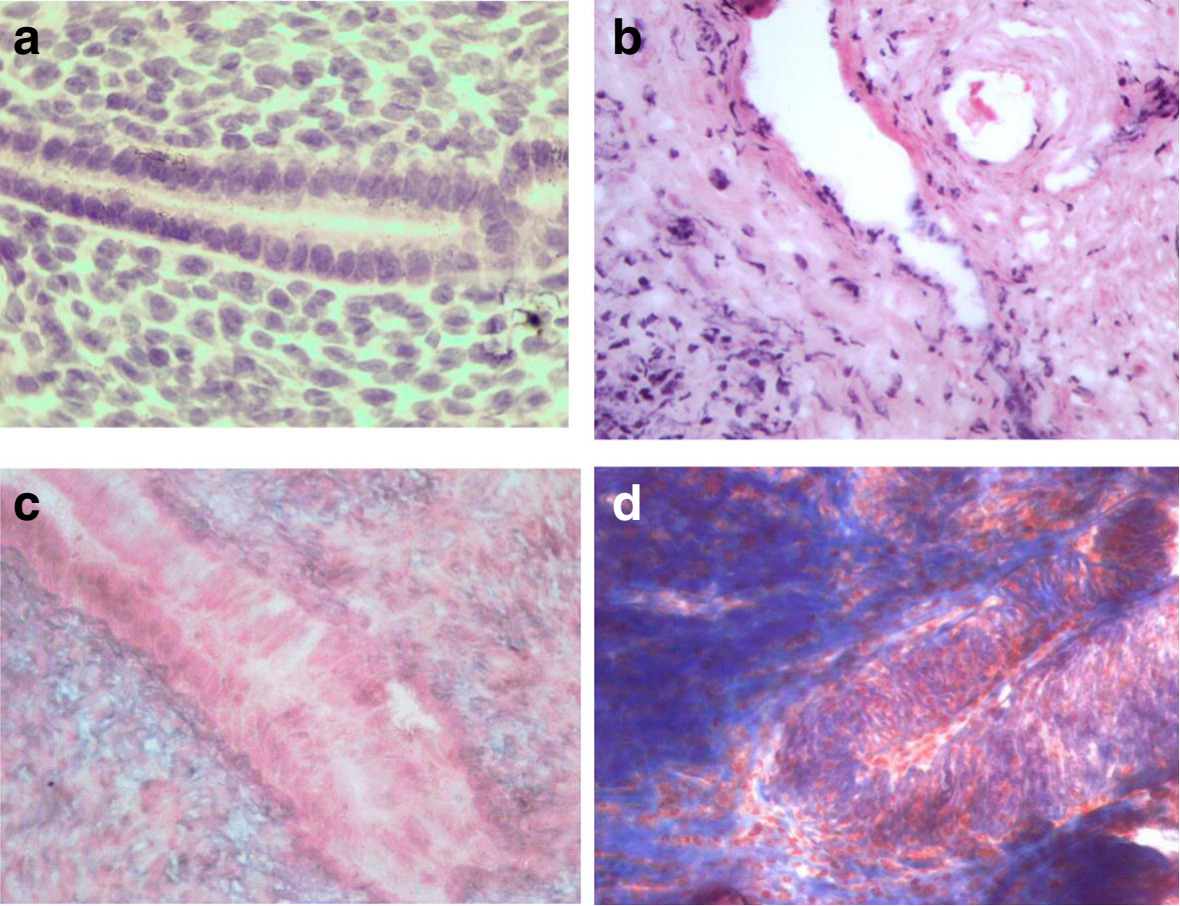
Histological assay of uterus injury in AS modeling rats. **a** Rat uterus without injury (H&E), **b** rat uterus three estrus cycles after injury (H&E); **c** rat uterus without injury (Trichrome staining), **d** rat uterus three estrus cycles after injury (Trichrome staining). Magnification: ×1000

Histologically, Asherman’s syndrome is indicated by fibrosis, sparsely glandular, inactive or cystic dilatation, and ischemic injury. In modeled rats morphological changes of endometrium were revealed by hematoxylin and eosin (HE) and Trichrome staining (TS) (Fig. 6). Histological evidence confirmed the development of post-traumatic fibrosis in the uterus. In normal control groups, the surface of endometrial cavity was covered by columnar epithelium with abundant endometrial glands (Fig. 6a). In the model animals, uterine endometrial glands were strongly reduced with tissue hyperplasia (Fig. 6b). TS demonstrated a lot of tightly arranged stromal blue collagen fibers in the model group (Fig. 6d).

### Pregnancy outcome and litter size in rats after eMSC transplantation

**Fig. 7:**
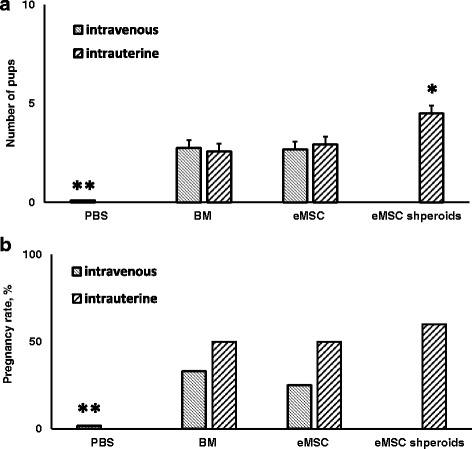
Number of pups (**a**) and pregnancy rate (**b**) in rats with modeled AS after intravenous or intrauterine transplantation of eMSC and rat BM. **a** **p < 0.01, animals received intravenous or intrauterine cell transplantation vs. PBS; *p < 0.01, eMSC spheroids vs. BM or monolayer eMSC transplantation. (Kruskal-Wallis H-test followed by Mann-Whitney *U* test with Bonferroni correction). **b** **p < 0.05, animals received intravenous or intrauterine cell transplantation vs. PBS. (Fisher’s exact test). *BM* rat bone marrow, *eMSC* endometrial mesenchymal stem cells

Rats with traumatized uterus modeling Asherman’s syndrome received cell therapy 72 h after the injury. To investigate the functional improvement in Asherman’s syndrome after cell transplantation, the female rats in the third estrous cycles after transplantation were mated to Wistar males for a period of 3 months. Rat bone marrow (BM) and eMSC were delivered intravenously or intrauterinely. eMSC spheroids were transplanted only into the uterus because of the pulmonary embolism risk after their intravenous administration. The therapeutic effect was evaluated by pregnancy outcome and litter size. The results of the cell administration via the tail vein or intrauterine are presented in Fig. 7. It is seen that the conception rate and litter size is very low in the animal group that received PBS injections (the control group of the Asherman’s model). Cell transplantation to animals with modeled Asherman’s syndrome improved their fertility (Fig. 7).

The most adequate measure of female fertility is the ability to give birth to pups. The average litter size was about three pups in all animals groups received eMSC from monolayer or rat BM cells either in tail vein or intrauterine. Their number increased to about five in animals treated with eMSC in spheroids delivered into the uterus (*p* < 0.01) (Fig. 7a). We did not observe the difference in therapeutic results using rat BM cells (autologous cells) and human eMSC from monolayer (heterologous cells). The therapeutic effect did not depend on the cell delivery route.

Figure 7b presents the conception rate in animals that received cell therapy. It is seen that control rats that received PBS injection were practically unable to conceive. The number of pregnant rats drastically increased after cell administration. The conception rate was higher with intrauterine administration then intravenous. It enhanced more with intrauterine delivery of eMSC in spheroids. However, the observed differences were not statistically significant.

## Discussion

MSC are a major cell source used for cell therapy [29]. Most frequently, these cells are derived from bone marrow, adipose tissue, umbilical cord blood, endometrium, and menstrual blood. Earlier, we obtained MSC from the menstrual blood (eMSC) [22]. eMSC meet the criteria of human multipotent MSC suggested by the International Society for Cellular Therapy [30]. They are adhesive to plastic under standard culture conditions, have fibroblast-like morphology, express CD146, CD140b, CD105, CD73, CD90, CD44, HLA-1, lack CD34, CD45, and HLA DR (class II), are multipotent, and able to differentiate into mesoderm lineages [22].

MSC hold promise in the treatment of different diseases. Numerous clinical trials of MSC application for cure of various tissue and organs disorders are in progress [31]. Unfortunately positive results of cell therapy were not achieved in some cases [29]. It is considered that aggressive conditions of damaged tissue leads to poor survival of transplanted cells and reduces the efficacy of stem cell therapy [19]. Cell preconditioning before transplantation that increases their survival and function is under intensive research.

In this study we tried to optimize cell-based therapy by the cell pretreatment prior to transplantation. Recent reports provide evidence that the aggregation of MSC into three-dimensional (3D) multicellular spheroids enhances the therapeutic potential of cells [32]. eMSC organized in spheroids retain all properties of eMSC in suspension: growth characteristics, differentiation potential, and expression of CD markers, except for CD146. It is known that CD146 expression in MSC may be heterogeneous and depends on the tissue and molecular environment. It has been demonstrated that CD146 is spontaneously downregulated with passaging at both mRNA and protein levels and that the high expression of CD146 reduces the proliferative self-renewal and osteogenic differentiation potential of dental MSC [33]. Another group of scientists found out that CD146^−/Low^ and CD146^High^ bone marrow MSC did not differ in number of colony-forming units-osteogenic, adipogenic, and chondrogenic differentiation or in vitro hematopoietic-supportive activity [34]. MSC assembled in spheroids have slightly higher expression of pluripotent genes Nanog and Sox-2 but not Oct4. Dedifferentiation and enhanced stemness seems to be a key feature of MSC spheroids and probably contributes to their improved therapeutic effect.

Cell therapy based on MSC transplantation is applied in clinic as a new treatment strategy for different diseases. Few attempts to use cell therapy for AS treatment are known. Bone marrow cells in combination with traditional AS therapy (surgical resection and hormonal therapy) gave positive results [35–37]. In these reports, bone marrow cells were not cultivated in vitro. However, cell therapy required a large cell biomass that can be reached by cell expansion in vitro. Recently, it was reported that autologous MSC derived from the menstrual blood and shortly cultivated in vitro significantly increased endometrial thickness in women with severe AS [38]. However, in some cases in patients with AS, obtaining of autologous biological material is impossible.

Cell therapy efficacy for AS treatment is actively tested on animal models. In the murine model of AS the transplantation of mouse bone marrow stem cells increased the number of animals able to give birth [8]. Stem cells isolated from rat adipose tissue combined with estrogen, appeared to be a highly effective alternative to induce regeneration of endometrium in rat model of AS therapy [39].

In our experiments on a rat model of AS we used human cells derived from the menstrual blood (eMSC) and suspension of rat bone marrow cells as control transplantation material. We found that eMSC from monolayer culture and rat bone marrow cells exhibit similar positive therapeutic effect estimated as pregnancy outcome and litter size. The therapy efficiency was slightly dependent on the route of the cell administration but intrauterine cell transplantation seemed more effective than cell injection into the tail vein.

The next step of our investigations was to improve the effect of therapy using preconditioned MSC. We found that intrauterine administration of eMSC organized in spheroids significantly improved fertility of rats with modeled AS. Why MSC spheroids exhibit higher therapeutic potential is not quite clear. Most eMSC in spheroids are in the quiescent state. It was reported that quiescent cells had higher survival rate under unfavorable conditions [24]. The comparison of the cell cycle distribution of eMSC spheroids and 2D eMSC prepared for transplantation showed that both cell populations had a similar pattern. MSC may have higher differentiation capacity [40]. We compared decidual differentiation of eMSC in monolayer and spheroids but did not find the difference between these cells. The common idea is that MSC spheroids have a role in tissue repair and regeneration via the secretion of soluble trophic factors that enhance the response of damaged tissues through paracrine activation [41]. Recent studies established that MSC did not engraft or differentiate to a large extent in sites of transplantation in vivo [42]. Frequently, therapeutic benefit was observed without evidence of engraftment. The cells enhanced tissue repair or limited tissue destruction by paracrine secretions [40, 42]. Indeed, in our experiments, synthesis of angiogenic and anti-inflammatory secreted factors drastically increased in eMSC organized in spheroids.

Taken together, our results show that transplantation of human MSC isolated from the menstrual blood (heterogonous cells) to Asherman syndrome rat models had the therapeutic effect similar to that observed after administration of rat bone marrow cells (autologous cells). Application of eMSC in spheroids significantly improved the rat fertility. It opens a new perspective for AS treatment with menstrual blood-derived MSC both autologous and heterologous. The idea is supported by the findings that intravenous and intrathecal transplantation of eMSC in four patients with multiple sclerosis did not reveal the treatment-associated adverse effects during more than 1 year of monitoring [43].

## Conclusions

Human endometrial stem cells (eMSC) can be successfully applied for Asherman’s syndrome (AS) treatment in the rat model. eMSC organized in spheroids were more therapeutically effective than the cells in monolayer. We suggest that improved therapeutic effect is realized via enhanced secretion of angiogenic and anti-inflammatory factors. After transplantation of eMSC in spheroids the pregnancy outcome and litter size in rats with AS was higher than in rats that received autologous rat bone marrow cells. It proposes possible application of heterologous eMSC for AS treatment in case of failure to use autologous cells.

## Abbreviations

AS: Asherman’s syndrome
BM: Rat bone marrow
eMSC: Endometrial mesenchymal stem cells
EP2: Prostaglandin E receptor
ESC: Human embryonic stem cells
HGF: Hepatocyte growth factor
IGFBP-1: Insulin-like growth factor binding protein-1
MSC: Mesenchymal stem cells
TSG-6: Tumor necrosis factor-α-induced protein 6
